# Intercomparison of photogrammetry software for three-dimensional vegetation modelling

**DOI:** 10.1098/rsos.172192

**Published:** 2018-07-11

**Authors:** Alexandra Probst, Demetrios Gatziolis, Nikolay Strigul

**Affiliations:** 1 Department of Biology, University of Washington, Seattle, WA, USA; 2 USDA Forest Service, Pacific Northwest Research Station, Portland, OR, USA; 3 Department of Mathematics and Statistics, Washington State University Vancouver, Vancouver, WA, USA

**Keywords:** remote sensing, photogrammetry, forest modelling, simulation, vegetation three-dimensionalre constructions, tree crown geometry

## Abstract

Photogrammetry-based three-dimensional reconstruction of objects is becoming increasingly appealing in research areas unrelated to computer vision. It has the potential to facilitate the assessment of forest inventory-related parameters by enabling or expediting resource measurements in the field. We hereby compare several implementations of photogrammetric algorithms (CMVS/PMVS, CMPMVS, MVE, OpenMVS, SURE and Agisoft PhotoScan) with respect to their performance in vegetation assessment. The evaluation is based on (i) a virtual scene where the precise location and dimensionality of objects is known *a priori* and is thus conducive to a quantitative comparison and (ii) using series of *in situ* acquired photographs of vegetation with overlapping field of view where the photogrammetric outcomes are compared qualitatively. Performance is quantified by computing receiver operating characteristic curves that summarize the type-I and type-II errors between the reference and reconstructed tree models. Similar artefacts are observed in synthetic- and *in situ*-based reconstructions.

## 1. Introduction

The advent of powerful workstations, cloud computing, inexpensive unmanned aerial systems (UASs) serving as image acquisition platforms and the evolution of pertinent algorithms [1] have made photogrammetry broadly available for many applications. Photogrammetry employed over natural scenes, including forested landscapes, is an emerging research area with potential to becoming a useful tool for spatially explicit environmental assessments [2–5]. A typical application relies on series of images, acquired with substantial field-of-view overlap, featuring the object(s) of interest and depicting them from a variety of viewing locations. The output of the photogrammetric processing is an abstraction organized in the form of a point cloud that represents the targeted object. The point cloud, frequently known as a three-dimensional scene or object reconstruction, can be used to quantify the distribution, volume and spatial extent of vegetation objects, and to complement spatially explicit individual-tree-based forest models [6–9]. A thorough evaluation of this emerging methodology is warranted, considering that the achieved reconstruction precision and completeness depends on many factors, such as the geometric and physical properties of the objects, illumination regimes, weather and the settings applied to the underlying algorithms.

Vegetation objects are among the most challenging for photogrammetry [2]. It has been well documented that the algorithms upon which three-dimensional reconstructions depend work best with images of objects that are completely stationary, solid, well-lit and have patterns or textures that are easily detected and distinguished from one another [10,11]. However, most vegetation objects including tree crowns are not solid, allow sunlight through them, can present with infinite background (e.g. sky when viewed laterally), and comprise branches, leaves and needles at divergent orientations and sizes. This geometric complexity ensures the absence of smooth surfaces, even at local spatial scales. The surface of a single leaf, the basic structural element of a deciduous tree crown, is easily discernible when viewed from a frontal perspective, but it often collapses into a negligible area in a lateral view. This and other similar idiosyncrasies of vegetation are a substantial challenge to many popular algorithms, which are based on gradient calculations, patch surface approximations and local patch expansions. Wind-induced variability in the relative placement of crown elements is an additional complication. As crowns are partially transparent and actively adsorb light, their light reflection properties tend to vary strongly by viewing location [2]. These phenomena inhibit the identification of key features common across different images and inflate the error embedded in recursive camera positioning calculations known as bundle adjustment. Thus, while the ground, buildings, stones, roads and other man-made objects are always represented nearly perfectly even in complex scenes, trees, shrubs and other vegetation elements tend to contain artificial discontinuities (holes). The quality of three-dimensional reconstructions in a forest setting also depends on the photogrammetric workflow and particular software selection. The motivation for this study has been the realization that alternative software applied to the same set of images results in three-dimensional models of notably different quality. While all algorithmic implementations across software packages are conceptually similar, their performances are not. Common artefacts include non-existent vegetation components and transposition of background objects such as clouds or terrain to foreground tree branches and leaves [2]. The frequency and magnitude of these artefacts varies among software packages.

Complete photogrammetric workflows are two-stage processes. The first step generates what is known as a sparse model, a low-density cloud comprising the locations of key scene features, each identified on a number of images. It also calculates the camera position corresponding to each image. The second step uses the information generated in the first to generate the dense model, the point cloud referred to as three-dimensional reconstruction. With few exceptions, the exact formulation of and settings applied to the algorithms used in generating the sparse and dense models is proprietary. In this study, we compare two software packages that do support sparse model generation, SIFT/Multicore Bundle Adjustment combination (packaged in VisualSFM) and PhotoScan, and a number of alternatives that support the derivation of dense models: CMPMVS, CMVS/PMVS (packaged in VisualSFM), MVE, OpenMVS, SURE and PhotoScan. The second group requires an existing sparse model formulation. Of the software considered here, only PhotoScan supports both processes. Performance was evaluated in two different settings: (i) a virtual reality scene where the dimensionality and locus of every structural scene element is known precisely and hence is conducive to a quantitative comparison, and (ii) actual, real-world scenes where reconstruction quality is evaluated visually. The virtual environment showcases a tree with realistic features, depicted in 200 high-resolution images rendered via Povray, an open-source ray-tracing software. The virtual tree images were processed with each of the aforementioned software. A quantitative assessment of reconstruction quality was obtained by computing receiver operating characteristic (ROC) curves that summarized the type-I and type-II errors between the reference and reconstructed tree models.

## 2. Material and methods

### 2.1. Software and three-dimensional reconstruction workflows

VisualSFM and PhotoScan, the two software packages that support sparse model generation, follow a similar approach. They detect image features using computer vision algorithms (e.g. SIFT [12], scale-invariant feature transform, and SURF [13], speeded-up robust features), compute similarity indices between image pairs using identified common features, and ultimately deduce the relative positioning (viewpoint) of each image within the natural scene. Images in violation of predetermined position consistency criteria are either removed or placed in separate clusters, each of which yields its own sparse model and features independent scale and coordinate system orientation. If requested, both software estimate the parameters of the classic Brown–Conrady camera lens distortion model and use it to obtain an adjusted, distortion-free version of each image. With the exception of PhotoScan, all other software packages used in this study to generate dense point cloud models relied on the sparse model and camera orientation obtained by using VisualSFM.

In addition to proprietary algorithm structure and settings, the software packages evaluated offer numerous, albeit often obscure customization options and parameters. VisualSFM, for example, uses an initialization (.*ini*) file where the user has the option to customize the values of a total of 76 parameters. A few of them control program execution such as whether to use hardware accelerators if available, enable remote control, disable asynchronous writing to temporary disk space or specify the number of threads to use. Others control the configuration of outputs, for instance, whether generated point clouds should be saved in text or binary format. Both parameter groups have descriptive names and, except the number of threads to use, accept binary (0/1), easy to deduce, values. The role of the remaining parameters and the range and type of values they accept can be particularly challenging to decipher even for a user well versed in the theory and idiosyncrasies of photogrammetric processing. Ambiguity and verbosity in parameter configuration is not limited to VisualSFM.

In this study, extensive evaluation of numerous parameter combinations revealed that among a large list, three types of parameters present in all software packages function as primary controls of the dense cloud reconstruction phase. The first of them controls the intensity of pixel sampling along epipolar rays that originate at each camera location. Details on ray delineation can be found in [2]. Examining every pixel along each ray is supposed to yield the highest reconstruction quality but at a high, usually very high, computation cost. Alternatively, one or more pixels can be skipped along each ray resulting in computational efficiency gains at the expense of an anticipated progressive reduction in scene reconstruction consistency. The second parameter sets the minimum number of different images a scene element must appear on before it is accepted as a legitimate object or object component. For a given level of field-of-view overlap between sequentially acquired images, increases in the minimum image number threshold decrease the probability of a scene component included in the dense cloud. Decreasing the threshold increases the probability of errors of commission. The third parameter is designed to restrict how far from the camera an identified scene element can be before it is included in the dense point cloud. Except for the minimum image number threshold, parameter value enumerations and scales vary across software packages. Unlike the other software packages, PhotoScan offers five distinct pre-parametrized settings, labelled as ‘quality’, ranked from lowest to highest. This is probably designed to relieve the user from the onus of delving into cryptic parameter enumeration issues. We deduced via experimentation that the default parameter values provided with each software package offer a balance between computational cost and point cloud fidelity, and appear to correspond to the medium quality setting of PhotoScan. Considering that an exhaustive investigation of the effects of each custom configuration is practically and logistically infeasible, we proceeded with using the default settings. Details are available in appendix D.

### 2.2. Imagery

#### 2.2.1. Unmanned aerial system-based aerial images.

The set of aerial images used is detailed in [2]. A small UAS equipped with a GoPro 3+ Black camera was programmed to follow a circular trajectory (20 m radius) around a 16 m tall deciduous tree at a constant 12 m above-ground elevation with the camera oriented towards the vertical middle of the tree. The UAS was moving at a constant speed and acquired 200 5 MB images during a windless day. The camera features an f/2.8 wide-angle lens placed in front of a 12-megapixel sensor. No permissions were required prior to conducting our fieldwork. Using unmanned aerial vehicle (UAV)-based, nadir looking imagery featuring sparse and low vegetation on flat land, Wu [14], the author of the VisualSfM software, documented that scene reconstructions obtained by using the generic image calibration model embedded into his software produced a macroscopically concave ground surface, an artefact attributed to imprecise image calibration. To avoid a similar propagation of artefacts, we first calibrated the camera used in this study with the efficient procedure described in the OpenCV image processing library [15], and then instructed both VisualSFM and PhotoScan to skip the generic image calibration process.

#### 2.2.2. Synthetic images.

A virtual reality scene was generated using the Persistence of Vision Raytracer [16] software, following the method described in [17]. The scene and image capturing algorithm were designed to mimic true field conditions. The synthetic tree featured a single trunk and numerous branches, with the ground patterned to imitate grass. We added multiple light sources to ensure the scene was free from directional shadows, yet contained dappled shadow effects characteristic of real illumination conditions. The number and spatial allocation of the rendering viewpoints were identical to those used to acquire the UAS-based images. The lens calibration parameters used to undistort the UAS-based aerial images were applied. To precisely align the synthetic scene to each dense reconstruction, a prerequisite for meaningful comparisons, eight reference targets represented as cubes were added to the virtual scene. Their bright colours and distinct designs facilitated effortless alignment between reference and reconstructed scenes. For both UAS-based and synthetic images, the field-of-view overlap between sequentially acquired images was approximately 90%.

### 2.3. Comparison of three-dimensional reconstructions

#### 2.3.1. Analysis of artefacts.

We used CloudCompare (http://www.cloudcompare.org/) and Meshlab (http://www.meshlab.net/), both freeware products featuring user-friendly graphical interface, to manipulate the point clouds and perform three-dimensional model analyses including alignment, rendering and artefact segmentation. We located artefacts by first aligning the point clouds to the reference and then calculating the nearest neighbour distances. Computed at every point of the derived cloud, the nearest neighbour metric provides a spatially explicit assessment of reconstruction quality. This assessment was especially effective for the synthetic scene, where the original, POV-Ray-generated three-dimensional model served as ground truth. The metric, however, is a one-sided evaluator, unable to penalize incomplete reconstructions where parts of the reference are absent (omission errors). Further, it detects localized discrepancies and not an overall error for the entire reconstruction. As such, it is not well suited to a quantitative ranking of reconstruction quality across different generating software packages. This limitation was resolved via ROC curve analysis.

#### 2.3.2. Receiver operating characteristic curves.

The ROC curve is a classic diagnostic test evaluation tool broadly used in medicine and other disciplines [18]. ROC curves convey discrete, tabulated 2 × 2 tests computed for a given threshold value and consisting of frequency values for two correct positive test diagnoses (true positive (TP) and true negative (TN)) or frequencies, and two incorrect test diagnosis (false negative (FN) and false positive (FP)). Similar to its use in medicine, ROC curve analysis enabled quantitative comparison of three-dimensional reconstructions against the control synthetic scene. For a given separation distance threshold, the presence or the absence of spatial correspondence between points in the control scene and the reconstructed clouds is translated to True/False Positive/Negative cases. Points in the reconstruction cloud within a given radius from any point in the control synthetic scene are labelled as TP, and those further apart as FP. Similarly, points in the synthetic scene are labelled FN or FP. An ROC curve is delineated by considering a continuum of radii (separation distance thresholds). By definition, every ROC curve passes through the graph origin ([0, 0] coordinates), given that for separation distance between reference and model equal to zero there are no TPs or FPs. It also passes through the [1, 1] graph coordinates when the separation distance threshold exceeds in magnitude the scene size. In a flawless reconstruction, the curve would pass through graph coordinates [0, 1], thanks to the presence of only TPs and no FPs. Close proximity of an ROC curve to this point is indicative of a precise, high-quality reconstruction model. We calculated and used the area under the curve (AUC) [18,19] as a quantitative metric suitable for our purposes. All calculations were performed using the R software (www.r-project.org).

## 3. Results

### 3.1. Artefacts in three-dimensional reconstructions

**Figure 1.**
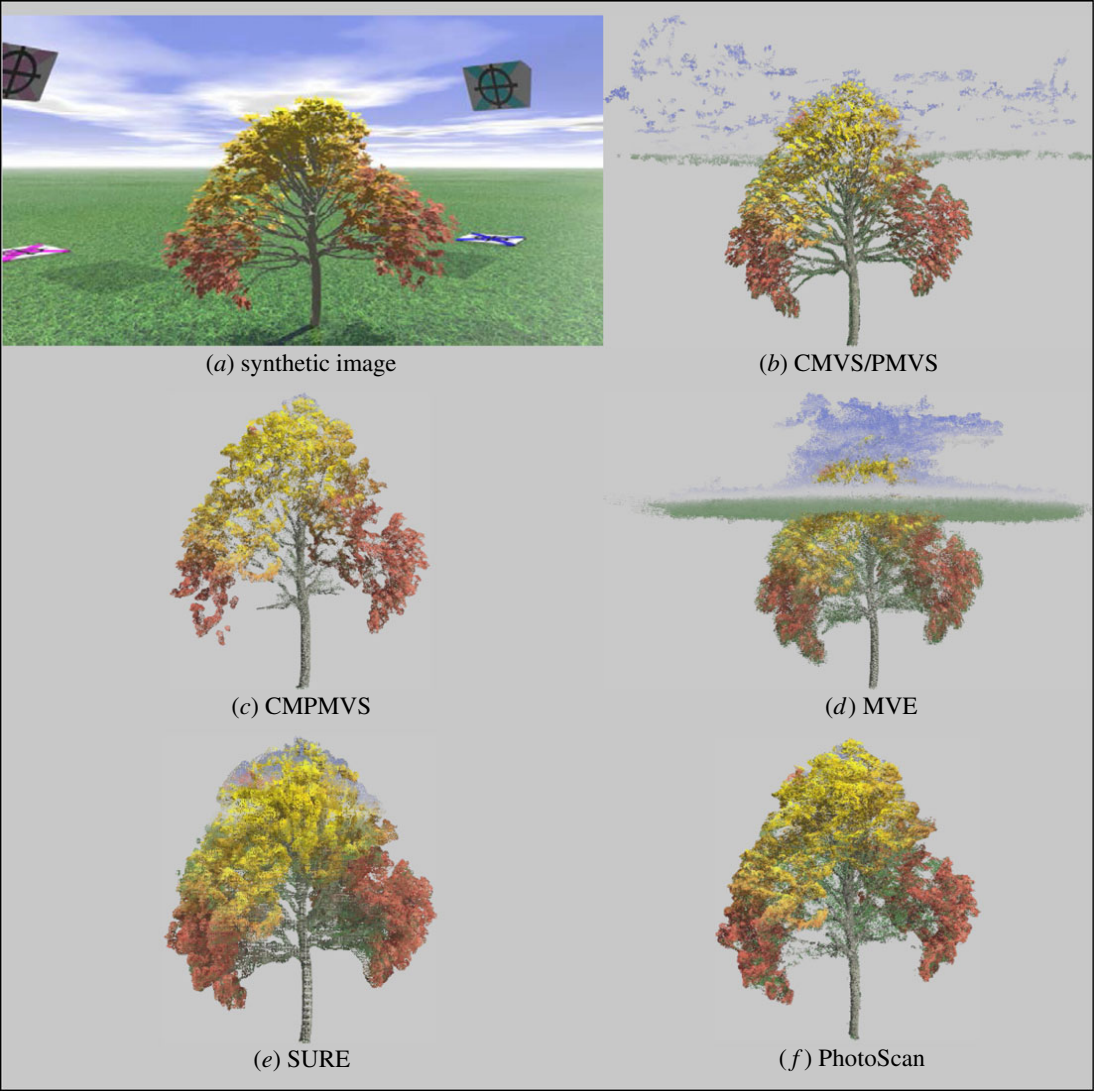
(*a*) Original POV-Ray model, (*b*–*f*) reconstructions by respective software.

**Figure 2.**
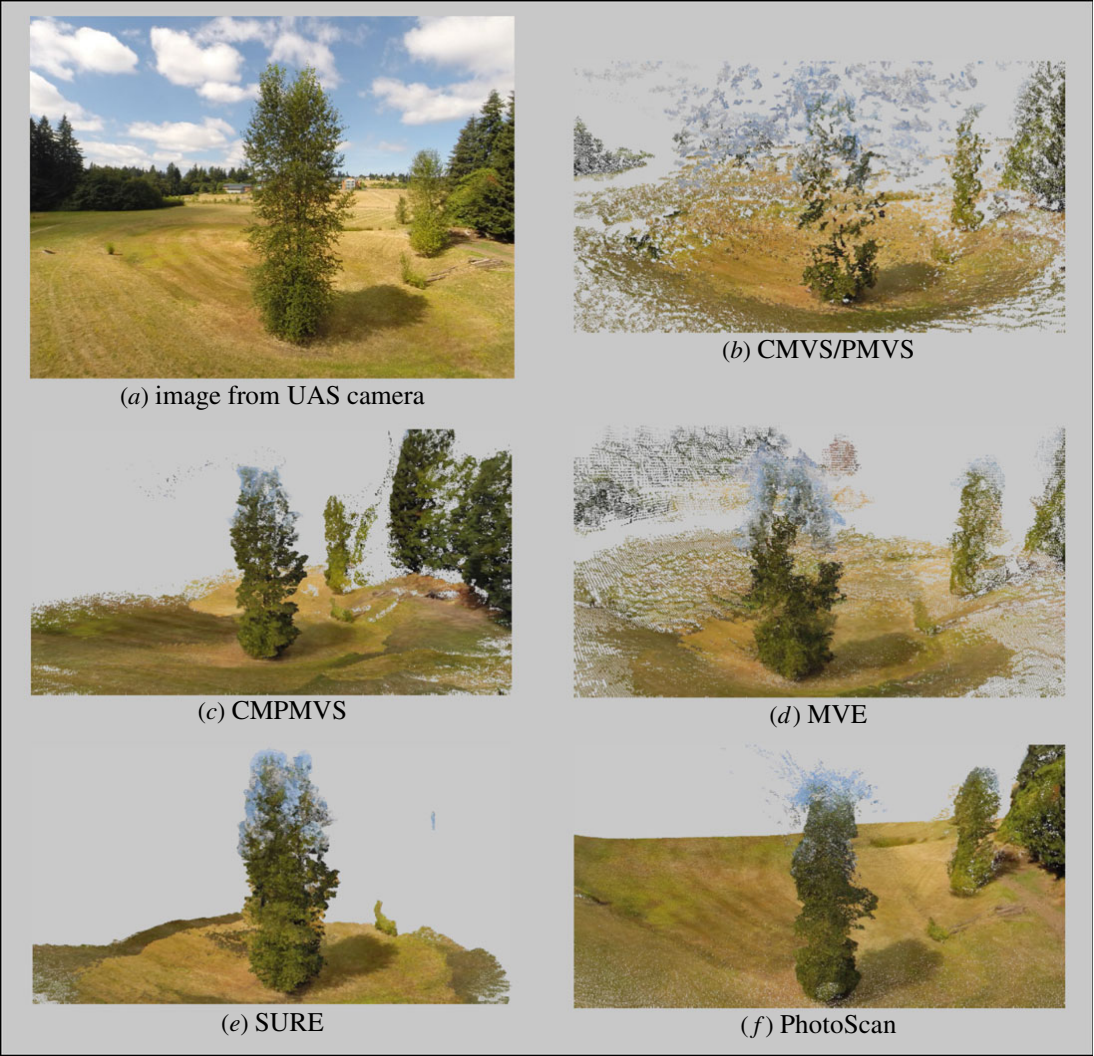
UAS-acquired scene image (*a*), and software-generated dense three-dimensional reconstructions (*b*–*f*).

**Figure 3.**
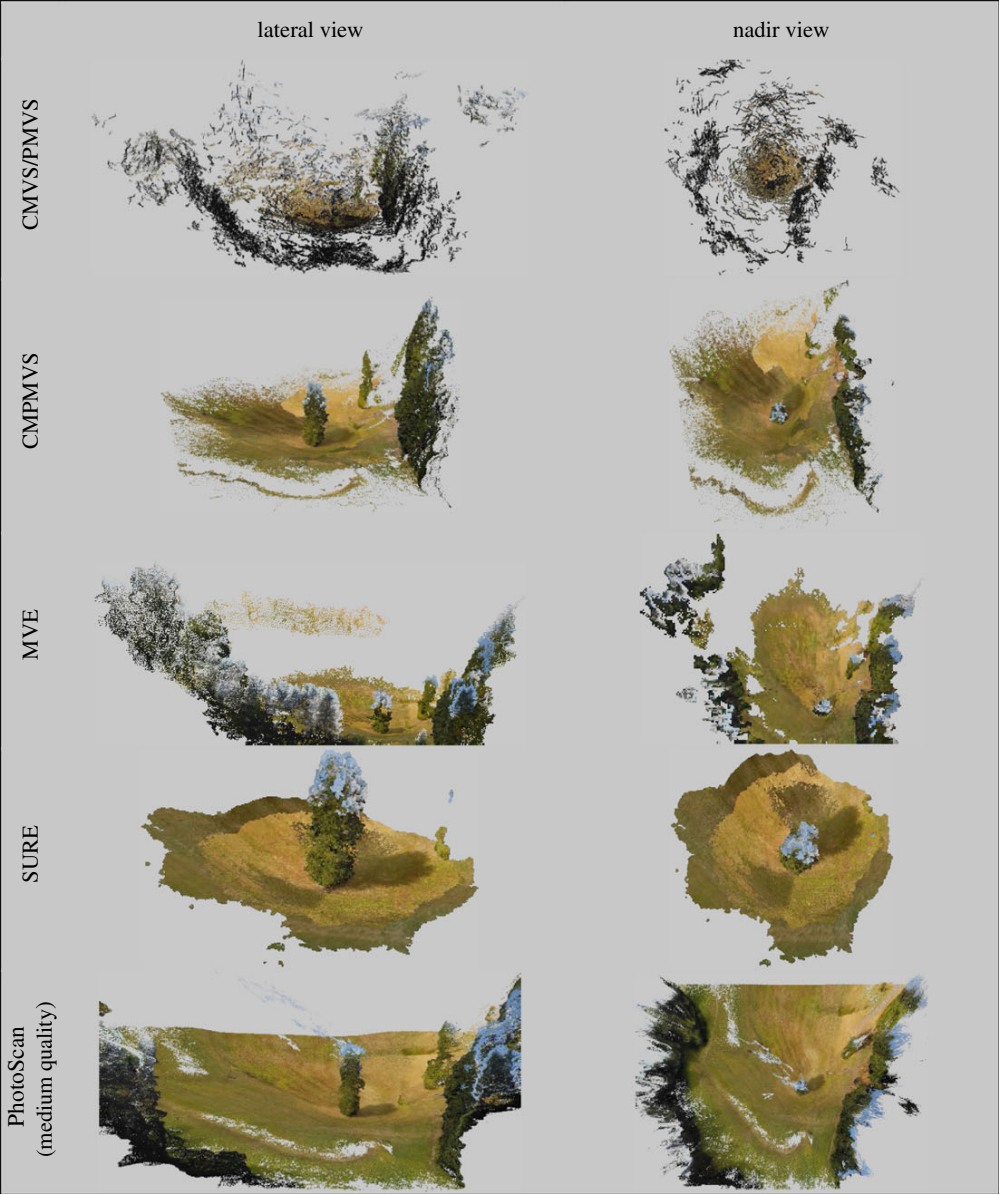
Lateral and nadir views of real scene reconstructions at varying scales.

While all software trials yielded object representations clearly identifiable as trees, each presented with inaccuracies and artefacts of variable frequency and magnitude (figures 1–3). The two most notable shortcomings observed were regions of the scene with vegetation present in the reference but void of points in the derived clouds (errors of omission) and artefacts which either floated in the sky or attached themselves to the trees and surroundings (errors of commission) (table 1 and figures 8–10). Incomplete representations, such as holes or discontinuities, of dominant scene object components are of decreased utility especially where they are expected to support dimensionality measurements. Floating artefacts obscure the scene and require laborious, subjective, and costly manual clean-up operations. We also encountered partial tree reconstructions, background scene elements attached to the foreground, discontinuities in the representation of the ground and in UAS-imagery-based reconstructions, distortions in the geometry of background scene components.

**Table 1.** Summary of artefacts in three-dimensional reconstructions.

software	floating artefacts virtual versus real scene	attached artefacts	partial reconstructions	background issues	ghosts
CMVS/ PMVS	150 / 411	few, thin layer of grass on synthetic tree branches	incomplete real tree, missing most of its upper half	tree and synthetic scene targets partially reconstructed	small pieces of real tree foliage reconstructed elsewhere
CMPMVS	39 / 58	few in synthetic scene. Large number of artefacts attached to the top of real trees	a few missing virtual tree branches	ground discontinuities	large sections of the synthetic tree reconstructed elsewhere in scene
MVE	1 / 7	sky attached on trees	large missing section in the upper middle of the real tree	object shape in real scene background deteriorates with distance	no ghosts
SURE	127 / 18	sky artefacts on upper parts of crowns, more pronounced in the real scene	complete tree reconstructions	practically no background in real scene	no ghosts
PhotoScan (lowest quality)	93 / 54	sky artefacts at the top of tree crown, larger in the real scene	complete tree reconstruction but hazy shape with hollow appearance	ground discontinuities, distorted background trees	no ghosts
PhotoScan (low quality)	35 / 70	small grass and sky artefacts on synthetic tree. Large sky artefacts in real scene	complete reconstruction of trees but somewhat hazy shape	ground discontinuities, distorted background trees	no ghosts
PhotoScan (medium quality)	6 / 24	thin layers of grass mixed in synthetic tree crown. Large upper crown artefact in real scene	complete reconstruction of trees	ground discontinuities	no ghosts
PhotoScan (high quality)	3 / 27	misplaced thin layers of grass and sky in synthetic scene. Small sky artefacts attached to upper parts of the real tree	complete reconstruction of trees except for selected branches	discontinuities in real scene ground. Missing parts of synthetic scene targets	no ghosts
PhotoScan (highest quality)	0 / 29	no synthetic scene artefacts, small layer of sky to the real scene tree	almost half of the synthetic tree is missing	discontinuities in real scene ground. Partially reconstructed ground.	no ghosts

#### 3.1.1. Floating and attached artefacts.

The frequency and point membership of floating or disconnected point clusters were identified using CloudCompare's connected components tool executed with a level 8 octree setting. Cluster frequency considered by itself, however, can be a deceptive evaluation metric. Of the 127 disconnected clusters in the synthetic scene reconstruction obtained by SURE, the vast majority pertained to small grass regions. The overall model had no commission artefacts. Unlike SURE, 148 of VisualSFM's 150 disconnected clusters represented the sky enveloped the tree, and obscured the scene. Conversely, the cloud obtained by applying PhotoScan ‘highest quality’ setting presented with no floating artefacts but suffered from pronounced errors of omission. The rate of FP points can be seriously inflated by the presence of attached, or connected point cluster, artefacts as evident in figure 2. All workflows had erroneously identified background regions, typically from the sky or ground, as tree components, but the severity of such commission errors varied. MVE was by far the worst performer and generated an artificial-looking horizontal ring comprising a large number of points and enveloping the upper half of the synthetic tree's crown.

#### 3.1.2. Partial reconstructions and background objects.

Reconstructions featuring pronounced discontinuities are inconsequential for ecological research, forest mensuration or natural resource assessment purposes. The majority of software succeeded in generating complete or almost complete reconstructions of the targeted trees. PhotoScan's ‘lowest’ and ‘highest’ quality settings had the measurably worst performance. With the ‘lowest’ quality setting, all major tree components were reconstructed, but the overall point density was very low and precluded meaningful point cloud post-processing. In the ‘highest’ setting, large parts of both the synthetic and real trees were missing. Considering that the processing time with the ‘highest’ quality setting is substantially longer than with the other settings, the prevalence of missing tree components seemed unexpected.

#### 3.1.3. Ghosts.

By this term, we refer to a single objects or object parts that appear in more than one instance in a point cloud. They are probably produced because of errors in the derivation of certain camera positions. CMPMVS replicated parts of the synthetic tree's main stem but not branches or foliage. The duplicated stem instance was accompanied by a separate, distinct shadow cast on the grass background. VisualSFM also generated ghosts albeit smaller in size compared with those from CMPMVS. Duplicates of large, solid objects such as the main stems of trees are easily discernible. Duplicates of foliage dispersed among tree crowns, however, are very difficult to identify, and can have ramifications on desired vegetation measurements, such as volume and area values. We were able to detect these in the VisualSFM-derived point clouds because they had distinct spectral features compared to their surroundings.

### 3.2. Receiver operating characteristic curve evaluation

**Figure 4.**
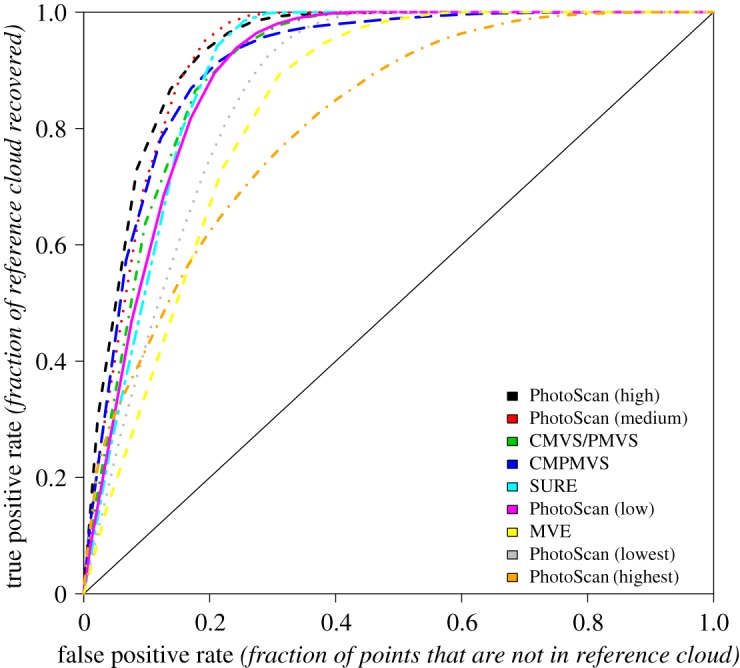
Software-specific ROC curves.

Computed ROC curves confirm that PhotoScan ‘highest’ quality, PhotoScan ‘lowest’ quality and MVE were inferior performers. The curves for the remaining packages were clustered, evidence that the respective reconstructions were of comparable, yet not equal quality. PhotoScan's ‘high’-quality setting produced the curve closest to the ideal [0, 1] graph point, with PhotoScan's ‘medium’ quality a close second. Curve ranks were not consistent across separation distance thresholds. For example, at smaller separation distances between reference and modelled scene, SURE performed worse than VisualSFM and CMPMVS, showing higher FP rates. At larger separation distances the curve ranking is switched and SURE is shown to be superior to both VisualSFM and CMPMVS (figure 4). Area under the curve (AUC) metric values (table 2) provide a quantitative ranking of software performance. They reveal three performance classes: PhotoScan ‘high’ and ‘medium’ as the top, CMVS/PMVS, CMP-MVS, SURE and PhotoScan ‘low’ as medium, with MVE, PhotoScan ‘lowest’ and PhotoScan ‘highest’ populating the low class.

**Table 2.** Area under the curve values per software package.

software	AUC
PhotoScan (high quality)	0.948
PhotoScan (medium quality)	0.947
CMVS/PMVS	0.937
CMPMVS	0.935
SURE	0.930
PhotoScan (low quality)	0.922
MVE	0.898
PhotoScan (lowest quality)	0.886
PhotoScan (highest quality)	0.822

## 4. Discussion

The pioneering work by Snavely *et al.* [20] was designed to accommodate any collection of digital images irrespective of origin, resolution and effective camera lens focal length or combinations thereof. Since then there has been a proliferation of proposed improvements, either novel or adaptations of pre-existing, analogue photogrammetry ideas. The set of software packages evaluated in this study are only a subset of a range of solutions available today. Considering the impetus provided by technological advancements and public interest in relevant applications, we expect further advancements to photogrammetric software.

A characteristic shared by the software packages examined is the absence of detailed technical documentation. For a few of them this issue is partially mitigated by online forums where users share experiences and ask for and provide feedback to processing challenges. While the information exchanged in these discussions can be valuable, it can also be speculative, subjective or applicable to a narrow set of conditions. In this study, we aimed at providing a detailed quantitative evaluation of performance at natural scenes.

In addition to the dearth of technical documentation, comprehensive sensitivity analysis with intent to optimize parameter values for a given set of UAS images is inhibited by the fact that photogrammetric processing, and dense cloud derivation in particular, is a very computationally intensive process. Based on our prior experience and the work performed in this study, we believe it is indeed possible, with a lot of effort and time investment, to occasionally improve on a structural attribute (completeness, positional accuracy, etc.) of a dense point cloud by trying combinations of values for the three primary controls mentioned in §2.1 instead of using the default values. However, the improvement is rarely substantial, regardless of whether the evaluation is visual or quantitative. Further, we have observed numerous cases where the parameter value combination proven to improve the dense point cloud of one scene has little effect on another similar scene.

This apparent absence of consistency is probably rooted to the fact that the concept of obtaining three-dimensional scene information using structure-from-motion techniques and the algorithms that support it have been designed for opaque objects with Lambertian (diffuse) or approximately Lambertian surface reflectance. Indeed, scenes comprising exclusively opaque objects tend to contain few artefacts. Similar behaviour is observed with point clouds generated from UAV-based imagery with nadir-oriented cameras over forested landscapes, a popular application [3–5]. In this configuration, the crowns of trees always have a terminal background, the forest floor and usually exhibit minute changes in solar illumination direction from one image to the next. In the viewing configuration of the real-world scene of this study, the tree crown background can be at infinite distance. The implication is that two neighbouring pixels positioned along an epipolar ray can be at markedly different distances from the camera. Besides, in two successive camera positions, one of them can be subject to direct solar illumination while the other is not, thanks, for example, to an intervening piece of foliage, leading to two images with very different overall brightness and contrast. Algorithms that anticipate only gradual changes in object parallax and illumination geometry, typical of opaque objects, fail to perform consistently for non-solid ones. Leaves behaving as nearly specular surface reflectors and of profiles that vary dramatically with changes in viewing geometry further compound the frequency and magnitude of artefacts.

Variability in the distance of tree crown components depicted in overlapping image regions from corresponding camera locations induces variability in representation scale. Owing to occlusion from crown components at the near end of the crown, components at the middle or far end may be visible only partially, even where they are positioned within the overlapping field of view of successively acquired images. Scale and occlusion rate variability paired with a high-quality setting specified by the user filter out scene components with representation frequency below the internal image number threshold. They thus lead to sizeable discontinuities or gaps and explain the high omission rates observed when using the ‘highest’ PhotoScan quality setting. SURE avoids this issue by excluding background scene components from the point clouds, while MVE follows the exactly opposite strategy. It prefers to deliver scene representations with larger spatial extent while accepting higher frequencies of artefacts and reduced point densities for background objects.

**Figure 5.**
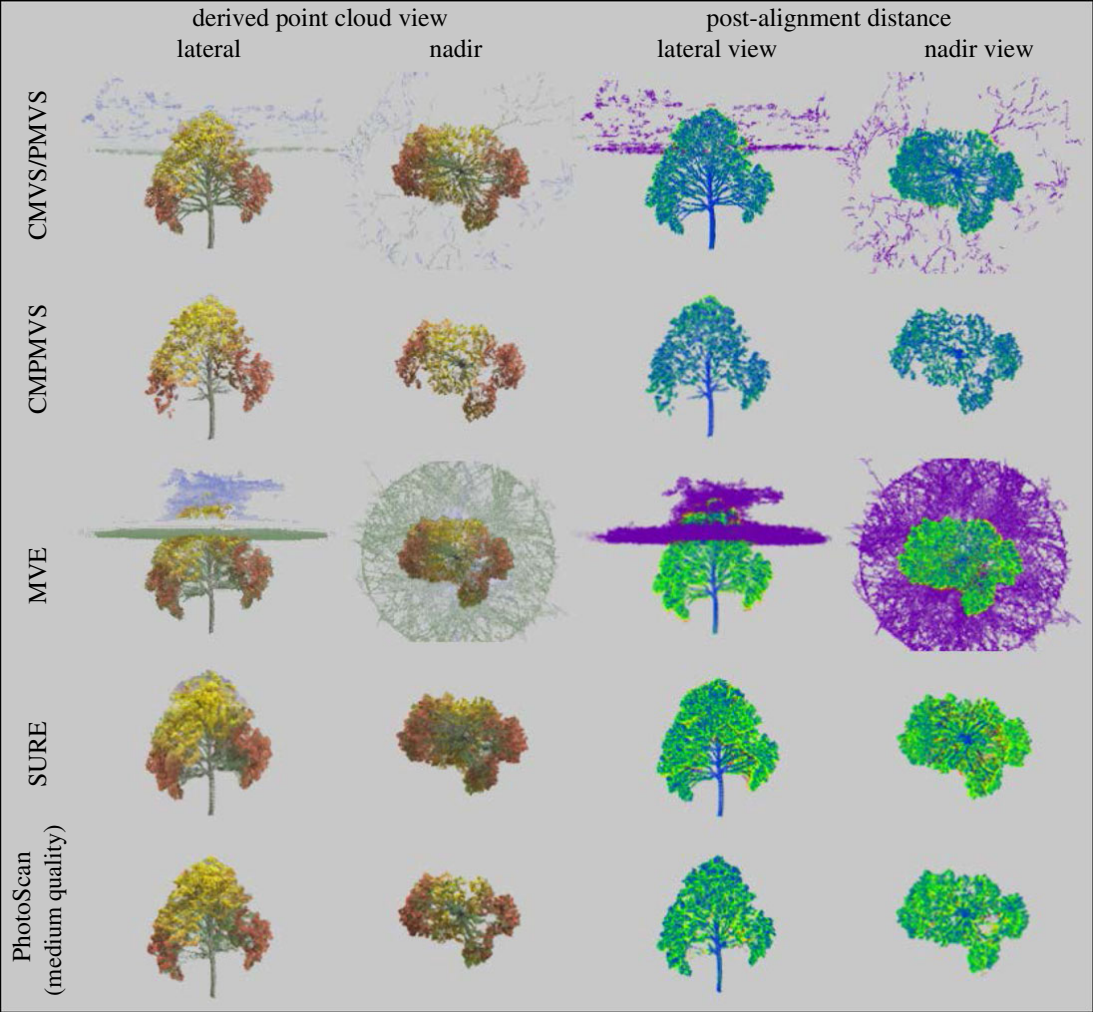
Software-derived point clouds aligned to reference synthetic tree in lateral and nadir views (first two columns) and coloured by classes of local distance discrepancy between reference and models (third and fourth columns). The class colouring scheme is blue for 0.0, green for 0.0075, yellow for 0.015 distance, red for 0.0225 and purple for larger distances (outliers). Distance values are relative to unit scene width.

**Figure 8.**
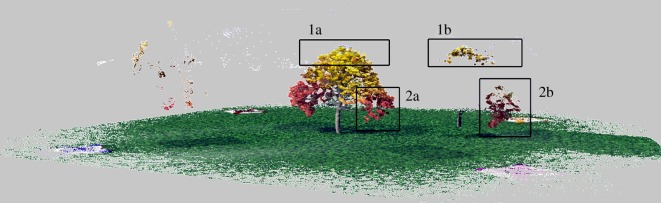
CMPMVS ghosts. 1b and 2b regions depict duplications of regions 1a and 2a, respectively.

**Figure 9.**
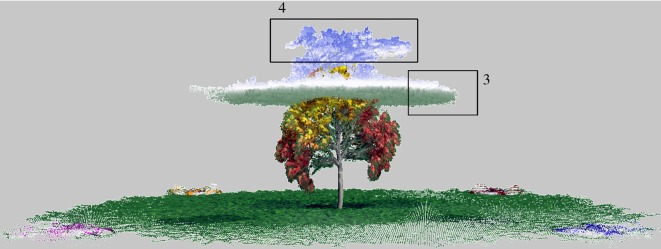
MVE attached artefacts. Region 3 shows a mixture of grass and sky enveloping the upper portion of the tree crown. Region 4 shows sky texture attached to the top of the tree.

**Figure 10.**
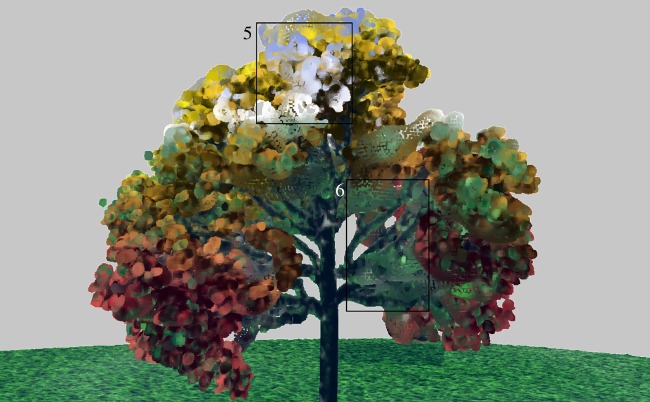
SURE artefacts. Region 5 shows upper crown leaves layered by points coloured as sky background. Region 6 shows the same phenomenon but this time of leaves and branches layered by points coloured as grass.

The apparent commission errors observed in the actual (figure 2) and synthetic scene (figure 5) for MVE and CMVS/PMVS can probably be reduced, if not completely removed, by masking on each image the regions representing the sky background. The masking operation can be accomplished by applying a combination of spectral and textural filters, given that a clear or cloudy sky has distinct digital pixel number ranges and texture from those of vegetation, ground or man-made objects. Even with this image preprocessing step, however, the upper portions of tree crowns will still inherit some of the sky's spectral signature, as foliage and vegetation material occupy only a portion of each pixel. Apparently, point cloud derivatives that capitalize solely on geometric attributes would not be affected by such colour-related artefacts.

For the rest of the tree crowns, simultaneously reducing the omission and commission artefacts in a systematic manner is probably infeasible, at least in the present state of software development. This is because in all software tested, the parametric configuration and application of pertinent algorithms appears to be static, in the sense that it does not adapt to local conditions. Enabling dynamic parametrization could be programmatically complex and further reduce processing efficiencies given that a second pass over the entire image set would be required, after the initial dense reconstruction is complete and the approximate structure of the scene is known.

It is suggested that for scenes dominated by crowns with complete and rigorous foliage along their entire vertical profile, the user specifies settings that require crown components to be present in a larger number of images, four or more, with processing of every other pixel along epipolar rays. Conversely, for more open, see-through crowns the minimum number of images required for scene component inclusion in the dense point cloud can be lower to avoid discontinuities in the dense point clouds generated.

The AUC metric computed from the ROC curves for the synthetic scene shows that the ‘high’ and ‘medium’ settings of PhotoScan, the commercial product, is performing better than all other software tested, probably thanks to embedded heuristics, but still contains notable artefacts. Whether the observed performance is significantly superior to that of CMVS/PMVS, CMPMVS and SURE, the freeware options, is not particularly clear. MVE and the other PhotoScan settings clearly have inferior performance. The same software ranking persists for the actual scene, although the evaluation in this case is purely visual. This software ranking presented assumes that all artefacts carry the same weight. In practice, the severity of artefacts can be specific to the type of information extracted from the point cloud. Missing a few isolated and small branches near the main stem of a tree, for example, can be unimportant when computing the volume occupied by tree foliage, but can be a serious shortcoming when assessing crown compaction ratios.

The qualitative and quantitative evaluation of photogrammetry-based three-dimensional representation of natural scenes presented here is, to our knowledge, the first such assessment. The framework described and the synthetic scene dataset made available here facilitate an expeditious and uncomplicated evaluation of software upgrades, primarily thanks to the utility of ROC curves and AUC metric. It should be reiterated that our assessment only applies to performance in reconstructing natural, outdoor environments using default settings. Owing to logistic constraints, the list of software evaluated is not exhaustive.

## 5. Conclusion

Photogrammetry-based analysis of vegetation structure is an emerging area of research. This work introduces an original and flexible approach for intercomparison of workflows and software, potentially useful for alternative scene compositions and application areas. It evaluates their ability to generate dense point cloud reconstructions of trees and shrubs. By including a synthetic, yet highly realistic scene with precisely known object dimensionality, it delivers a detailed, quantitative assessment of software performance. Study findings confirm that the same set of UAV-based images, or synthetic alternatives, processed with different software implementations of the structure-from-motion concept yield point clouds with different spatial characteristics. Findings suggest that the commercial software evaluated has slightly superior performance compared to freeware alternatives but scene representation completeness and positional accuracy does not improve monotonically with increases in processing complexity and execution time. Our findings pertain to vegetation structure and scene illumination conditions similar to those used in this study. Additional investigations would be needed prior to claiming applicability to other conditions. The methodology presented can serve as a guide to forest inventory specialists and analysts interested in obtaining detailed, three-dimensional representations of trees present in field plots economically, following an established road map.

## Figures and Tables

**Figure 6.**
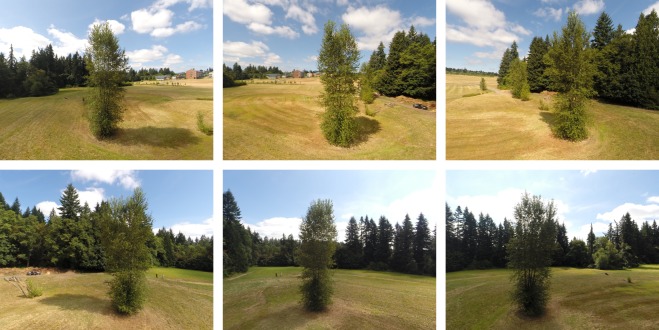
UAV-acquired photographs of the targeted tree and surrounding area from various viewing points along the platforms trajectory.

**Figure 7.**
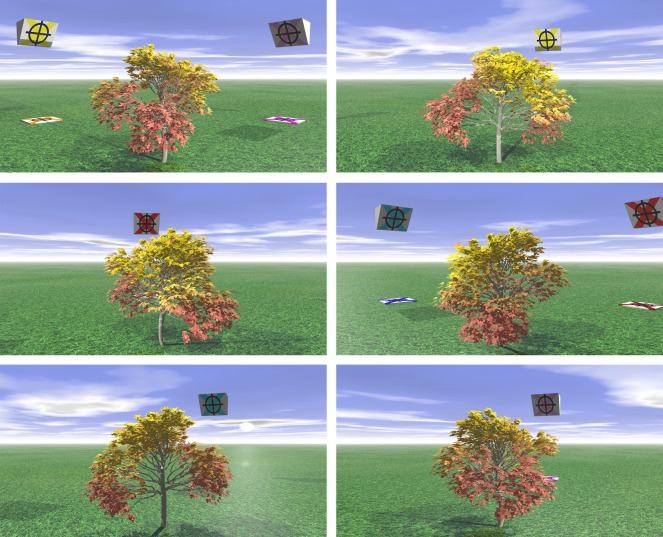
Images of the synthetic scene acquired at various viewing points around the targeted tree.

**Figure 11.**
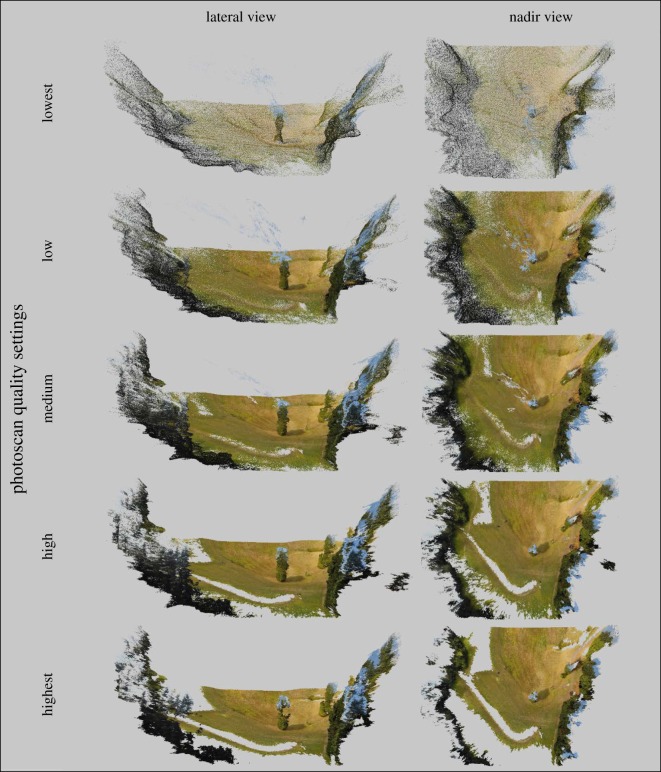
Lateral and nadir views of real scene reconstructions obtained with different quality setting of PhotoScan software.

**Figure 12.**
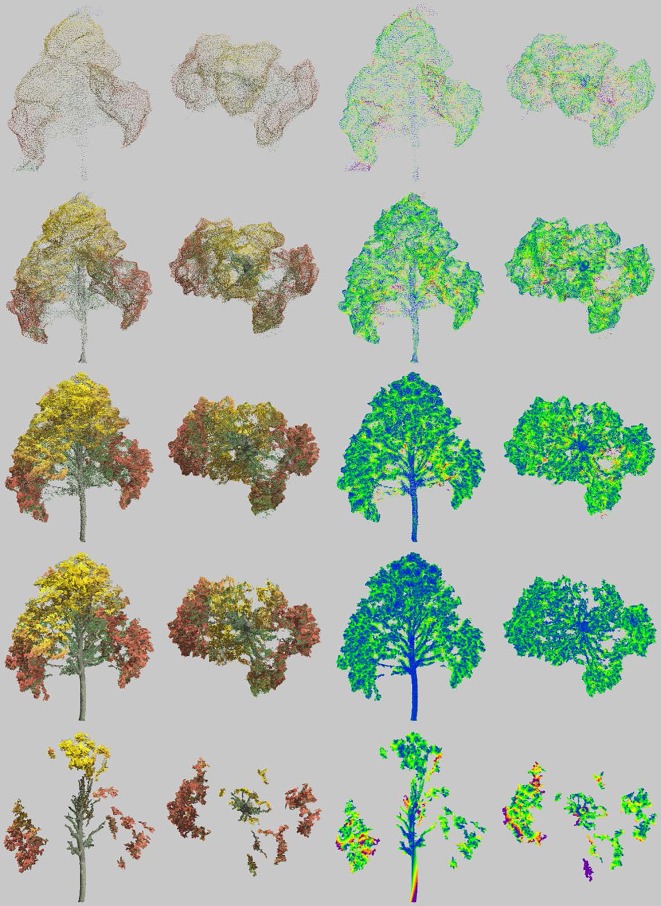
PhotoScan-derived point clouds with different quality settings, aligned to reference synthetic tree in lateral and nadir views (first two columns) and coloured by classes of local distance discrepancy between reference and models (third and fourth columns). The class colouring scheme is blue for 0.0, green for 0.0075, yellow for 0.015 distance, red for 0.0225 and purple for larger distances (outliers). Distance values are relative to unit scene width.

## Data accessibility

Original aerial UAS-based and synthetics imagery data used for the comparison of photogrammetric algorithms are available on the Dryad Digital Repository: http://dx.doi.org/10.5061/dryad.2459s12 [21].

## Appendix A. Software and workflow details

*VisualSFM*. VisualSFM is a three-dimensional reconstruction GUI developed by Changchang Wu [14]. CMPMVS, OpenMVS, SURE and VisualSFM's own dense reconstruction rely upon VisualSFM or similar structure-from-motion programs such as Bundler [20] to initially detect key points among the images. VisualSFM performs this task using its compute missing matches application. This application depends upon Wu's pre-emptive feature matching which first identifies top-scale features and focuses computational efforts on these image pairs [22]. Following this, a sparse reconstruction is computed from the key points and their camera orientations. VisualSFM computes sparse reconstructions efficiently via CPU and GPU parallelization using its SiftGPU and multicore bundle adjustment routines [23,24]. We used VisualSFM to generate the initial sparse models of both virtual and actual trees, which were then used for ensuing reconstructions by the software mentioned. Additionally, VisualSFM offers its own dense reconstruction option which we included in our comparisons. It computes dense reconstruction with Y. Furukawa's PMVS/CMVS module, which is based off his cluster views for multi-view stereo software [25,26] (table 3).

**Table 3.** Software and workflow details.

software	workflow	software output	interface	version	developers
VisualSFM	feature matching, sparse recon., dense point cloud	image orientation, dense point cloud	command line, GUI	0.5.25	C. Wu
CMPMVS	depth map, dense point cloud, mesh recon.	mesh	command line	0.6.0	M. Jancosek, T. Pajdla
MVE	depth map, dense point cloud, floating surface recon., mesh cleaning	image orientation, dense point cloud, mesh	command line, GUI	05/2016	S. Fuhrmann, F. Langguth, M. Goessele
OpenMVS	dense point cloud, mesh recon., mesh refining, mesh texturing	mesh	command line	0.7	Git-hub user cdcseacave
SURE	depth map, dense point cloud, mesh	mesh	command line, GUI	0.0	M. Rothermel, K. Wenzel
PhotoScan		image orientation, dense point cloud, mesh	command line, GUI	1.3.1	Agisoft LLC

*CMPMVS*. CMPMVS was developed by Michal Jancosek and Tomas Pajdla. It is a multi-view reconstruction software specifically designed to reconstruct weakly supported surfaces, such as transparent glasses or obscured ground planes [27]. It requires *a priori* known camera positioning and orientation information, in our case supplied by VisualSFM. Using a plane sweeping algorithm, CMPMVS creates a depth map for each image, which is then used to create a point cloud and finally a three-dimensional mesh. We implemented CMPMVS using the default parameters set in the batch file provided.

*MVE*. Researchers Simon Fuhrmann, Fabian Langguth and Michael Goessele created the reconstruction software pipeline known as Multi-View Environment (MVE) [28]. Like VisualSFM, MVE contains software for the complete reconstruction pipeline. However, we chose to use VisualSFM's sparse reconstruction in order to maintain consistency across comparisons.

*OpenMVS*. OpenMVS is a recently released open-source library aiming to provide a complete set of dense reconstruction algorithms (http://cdcseacave.github.io/openMVS/). OpenMVS creates a dense reconstruction and a mesh and furnishes the mesh surfaces with texture. At the time of this study, despite our best efforts, we could not manage to obtain reconstructions of the virtual reality environment with a quality consistent to the other workflows, and decided to omit this program from the comparisons.

*SURE*. SURE is a three-dimensional reconstruction software developed by Mathias Rothermel & Konrad Wenzel [29]. It is not an open-source program but provides licences for academic use. At SURE's core is the LibTSgm library, which contains modules that perform image triangulation from camera parameters. SURE requires a sparse reconstruction input, and accepts many forms including VisualSFM's nvm file.

*Agisoft PhotoScan*. Agisoft PhotoScan is a commercial three-dimensional reconstruction software produced by Agisoft LLC [30]. It can be used under commercial and educational licensing. PhotoScan is an all-in-one three-dimensional photogrammetry software which handles the entire modelling process from feature matching to dense reconstruction.

## Appendix B. Imagery datasets for model comparison

See figures 6 and 7.

## Appendix C. Artefacts in three-dimensional models

See figures 8–10.

## Appendix D. Different quality settings in PhotoScan.

See figures 11 and 12.
