# Correction to: ‘Intercomparison of photogrammetry software for three-dimensional vegetation modelling’

**DOI:** 10.1098/rsos.181297

**Published:** 2018-08-22

**Authors:** Alexandra Probst, Demetrios Gatziolis, Jean Lienard, Nikolay Strigul

*R. Soc. open sci.* **5**, 172192. (Published 12 June 2018). (doi:10.1098/rsos.172192)

The authors have agreed among themselves that Jean Lienard should also be identified as a co-author of the work. Thus, the author list now reads: Alexandra Probst, Demetrios Gatziolis, Jean Lienard and Nikolay Strigul.

Furthermore, the published work's ‘Authors’ contributions’ statement is now amended to read as follows:

Authors' contributions. A.P., D.G. and N.S. equally participated in the design of the study and wrote the manuscript. A.P. has conducted three-dimensional reconstructions. J.L. designed and conducted the quantitative comparison. All authors gave final approval for publication

Jean Lienard thanks his colleagues for agreeing this amendment.

